# Simulated biomechanical performance of morphologically disparate ant mandibles under bite loading

**DOI:** 10.1038/s41598-023-43944-8

**Published:** 2023-10-06

**Authors:** C. L. Klunk, M. A. Argenta, F. B. Rosumek, S. Schmelzle, T. van de Kamp, J. U. Hammel, M. R. Pie, M. Heethoff

**Affiliations:** 1 Graduate Program in Ecology and Conservation, Universidade Federal do Paraná, Centro Politécnico, Av. Cel. Francisco H. dos Santos, 100 - Jardim das Américas, Curitiba, PR 81531-980 Brazil; 2 Animal Evolutionary Ecology, Technische Universität Darmstadt, Schnittspahnstr. 3, 64287 Darmstadt, Germany; 3 Department of Civil Construction, Universidade Federal do Paraná, Curitiba, PR Brazil; 4 Department of Ecology and Zoology, Universidade Federal de Santa Catarina, Florianópolis, SC Brazil; 5 Institute for Photon Science and Synchrotron Radiation (IPS), Karlsruhe Institute of Technology (KIT), Eggenstein-Leopoldshafen, Germany; 6 Laboratory for Applications of Synchrotron Radiation (LAS), Karlsruhe Institute of Technology (KIT), Karlsruhe, Germany; 7 Institute of Materials Physics, Helmholtz-Zentrum Hereon, Geesthacht, Germany; 8 Biology Department, Edge Hill University, Ormskirk, Lancashire UK

**Keywords:** Biomechanics, Entomology

## Abstract

Insects evolved various modifications to their mouthparts, allowing for a broad exploration of feeding modes. In ants, workers perform non-reproductive tasks like excavation, food processing, and juvenile care, relying heavily on their mandibles. Given the importance of biting for ant workers and the significant mandible morphological diversity across species, it is essential to understand how mandible shape influences its mechanical responses to bite loading. We employed Finite Element Analysis to simulate biting scenarios on mandible volumetric models from 25 ant species classified in different feeding habits. We hypothesize that mandibles of predatory ants, especially trap-jaw ants, would perform better than mandibles of omnivorous species due to their necessity to subdue living prey. We defined simulations to allow only variation in mandible morphology between specimens. Our results demonstrated interspecific differences in mandible mechanical responses to biting loading. However, we found no evident differences in biting performance between the predatory and the remaining ants, and trap-jaw mandibles did not show lower stress levels than other mandibles under bite loading. These results suggest that ant feeding habit is not a robust predictor of mandible biting performance, a possible consequence of mandibles being employed as versatile tools to perform several tasks.

## Introduction

Morphological variation is a central component of biological evolution. Among arthropods, insects are taxonomically diverse and show substantial morphological variation associated with several aspects of their life-histories, including their feeding habits^1^. Insect mouthparts are ectognathus, meaning they articulate externally with the head and are composed of the labrum, a hypopharynx, a pair of mandibles and maxillae, and a labium^2^. Morphological diversification of insect mouthparts is associated with many insect feeding habits, enabling them to feed on food items with variable mechanical demands^3^. One crucial aspect of insect feeding is the ability to capture prey and chew food items using mandibles^4^, which leads to considerable mandibular morphological divergence across species^3^, and whose relevance is highlighted by the size of the mandible adductor muscles^2^.

Many current insect lineages exhibit a dicondylic pattern of mandible articulation with the head^4,5^. Dicondylic mandibles articulate with the head through two ball-and-socket joints, reducing mandibular movement to a single axis of rotation so that abduction and adduction are the only possible movements, in contrast to the ancestral condition of insects that were also able to protract their mandibles^4,5^. Despite a simplification in mandibular movement, this condition increases the stability of mandibular movement^5^, and insects with dicondylic mandibles exhibit a vast diversity of feeding habits and mandible morphologies^3^. While mandibular morphological variation is often associated with functional aspects, such as feeding characteristics^6,7^, there is no compelling evidence that such an association is a frequent evolutionary pattern^4,8^. In many insect lineages, the mandibles perform several functions, which compromises specialization and may underline the lack of association between mandible morphology and functional aspects. This is particularly true for ants, especially the worker caste^9,10^.

Ants are eusocial insects that exhibit a reproductive division of labor. Winged members of the colony, namely queens and males, are responsible for reproduction, while the wingless individuals, or workers, perform the non-reproductive tasks essential for colony maintenance^11,12^. To carry out their daily tasks workers rely heavily on their mandibles, which enable them to bite, transport objects, dig, process food, and perform other functions^9^. The significant reliance on mandibles for task performance has led to the crucial enlargement of the mandibular adductor muscle *M. craniomandibularis internus* (*0md1*) in workers, occupying most of the head’s internal volume. A much smaller pair of muscles, the *M. craniomandibularis externus* (*0md3*), is responsible for mandible abduction^13–19^. Due to mandible movement limitations, ant workers must perform various tasks by modulating the force and velocity of their bites, along with employing other mouthparts to further process food, for example. This modulation is accomplished through the versatility of the *0md1* muscle, composed of several sets of fibers that vary in their contractile characteristics^20,21^. Some muscle fibers have long sarcomeres, which generate slow but powerful contractions, whereas others consist of short sarcomeres that produce fast but weaker contractions, and the proportion of these distinct fiber types in the *0md1* also varies interspecifically^13^. In some specialized ant species, power amplification mechanisms have evolved in which subtle morphological modifications, mainly in the mandible articulations with the head, generate a mechanical system where the contraction of the *0md1* muscle stores potential energy that is suddenly released to generate high-speed strikes. This results in faster movements than possible under muscle contraction only^22–28^.

Beyond muscular modulation, bite performance can potentially be influenced by mandible morphology. While most ant mandibles are shovel-shaped with a narrow base and a triangular blade^29^, there is considerable interspecific variation in mandibular morphology^9^, which may reflect functional adaptations to specific feeding habits or other ecological roles^30^. Although some studies did not find a clear relationship between mandible shape and function^31^, there is evidence that particular ant species evolved specialized mandible shapes for specific tasks. For example, *Melissotarsus* ants have mandibles specialized for excavation^32^, while *Pheidole* spp. major workers have mandibles with a notable ability to exert pressure during a bite^33^. In leaf-cutting ants, the mandible shape of some worker types excels at cutting leaves^34,35^. Sub-major workers of *Eciton* spp. have mandibles that allow for a more powerful grip to carry large prey^36,37^. The falcate mandibles of *Dorylus* spp. males seem associated with a better capacity to grip the queen during copulation^18^. Ants with power amplification mechanisms, such as trap- and snap-jaw ants, have particularly distinctive mandibles that are longer and narrower than the remaining ants. Among many functional aspects, this may be an adaptation to withstand the mechanical demands of their powerful strikes^26,27,38,39^ and facilitate prey capture^30,39^. Additionally, the fossil record suggests that disparate mandible shapes were prevalent from the beginning of ant diversification^40^, and shovel-shaped mandible may not be the ancestral condition in ants^29,41^. Some authors argue that the ancestral ant may have relied on a falcate-shaped mandible^18^. Overall, understanding the functional implications of mandibular morphological variation in ants is a crucial area of research that can shed light on the ecology and evolution of these insects.

In addition to interspecific variation, mandible shape can vary intraspecifically between worker types in polymorphic ant species. In monomorphic ants, there is subtle morphological variation in the worker caste, mainly related to variation in body size^42,43^. In other cases, however, allometric scaling results in two or more recognizable discrete worker types (species often called dimorphic, trimorphic, and so on) or even continuous variation along a gradient, representing different levels of worker polymorphism^44^. Worker polymorphism can improve colony division of labor and task efficiency by allowing different worker types to specialize in distinct colony tasks^44,45^. There is evidence that variation in the mandible morphology of polymorphic workers is associated with task activity in some ant species^26,33–35^.

Biomechanical simulations are valuable tools to explore the relationship between mandible morphology and biting performance^8,26,33,47–50^. In recent years, there has been a significant increase in the availability of high-quality morphological data for ants due to improved access to microtomography facilities (high-resolution desktop and synchrotron µCT)^51^. It has allowed for a broad application of biomechanical simulations and studies of functional morphology, and has been particularly useful in investigating the relationship between mandible morphology and bite performance^52,53^. However, in the case of ants, most studies have focused on a few species^26,33,38,54^.

In this context, our study aimed to investigate the role of mandible shape in bite performance across a wide range of ant species. By biting performance we considered the distribution of stress in solid models of ant mandibles under loading conditions that characterize distinct biting behaviors. Stress is the force that concentrates in a specific area^55^. It is a mechanical response of the structure to external loading demands and is a valuable property to consider when comparing the responses of different organisms to similar external loading conditions^56,57^. We selected ants with different feeding habits, including generalist and specialist predators (trap-jaw ants), omnivorous ants, and one leaf-cutting species. The main focus of our study was to discuss how the variation in mandible morphology influences stress distribution and investigate whether predatory ants, which need to capture and subdue living prey, possess mandibles morphologically specialized in dealing with bite loading. To achieve this, we used Finite Element Analysis (FEA) to simulate biting behaviors in 3D mandible models of several ant species. Being widely applied in distinct engineering fields and more recently in the context of biological functional mechanics, FEA is a numerical technique used to approximate the mechanical responses of structures to the loading demands they need to withstand^56,57^. To be performed, FEA demands a digital representation of the structure of interest, knowledge of its material properties, and the loading conditions intended to be simulated^56,57^.

Our main hypothesis was that predatory species would show proportionally lower stress levels than non-predatory species in biting simulations, assuming that the need to capture and subdue living prey with the mandibles represents higher mechanical demands than dealing with dead organisms, plant tissues, or liquid exudates. We also expected that trap-jaw ants would perform better at strike biting than all other species since the power amplification mechanism of those ants allows for the generation of remarkably fast strikes^58,59^, whose mechanical demands need to be withstood by their mandibles. Additionally, we predicted that sturdier mandibles, such as those of major workers in polymorphic species like *Pheidole* spp., would perform better than slender mandibles in simulations of pressure biting, regardless of the main feeding habit of the species, due to their more robust constitution that allows for a better concentration of stresses around thicker regions of the mandibles^33^. By conducting this comparative study across a wide range of ant species, we aimed to improve our understanding of the relationship between mandible morphology and bite performance and shed light on the functional implications of mandible variation in ants.

## Results

### Colour maps

**Figure 1:**
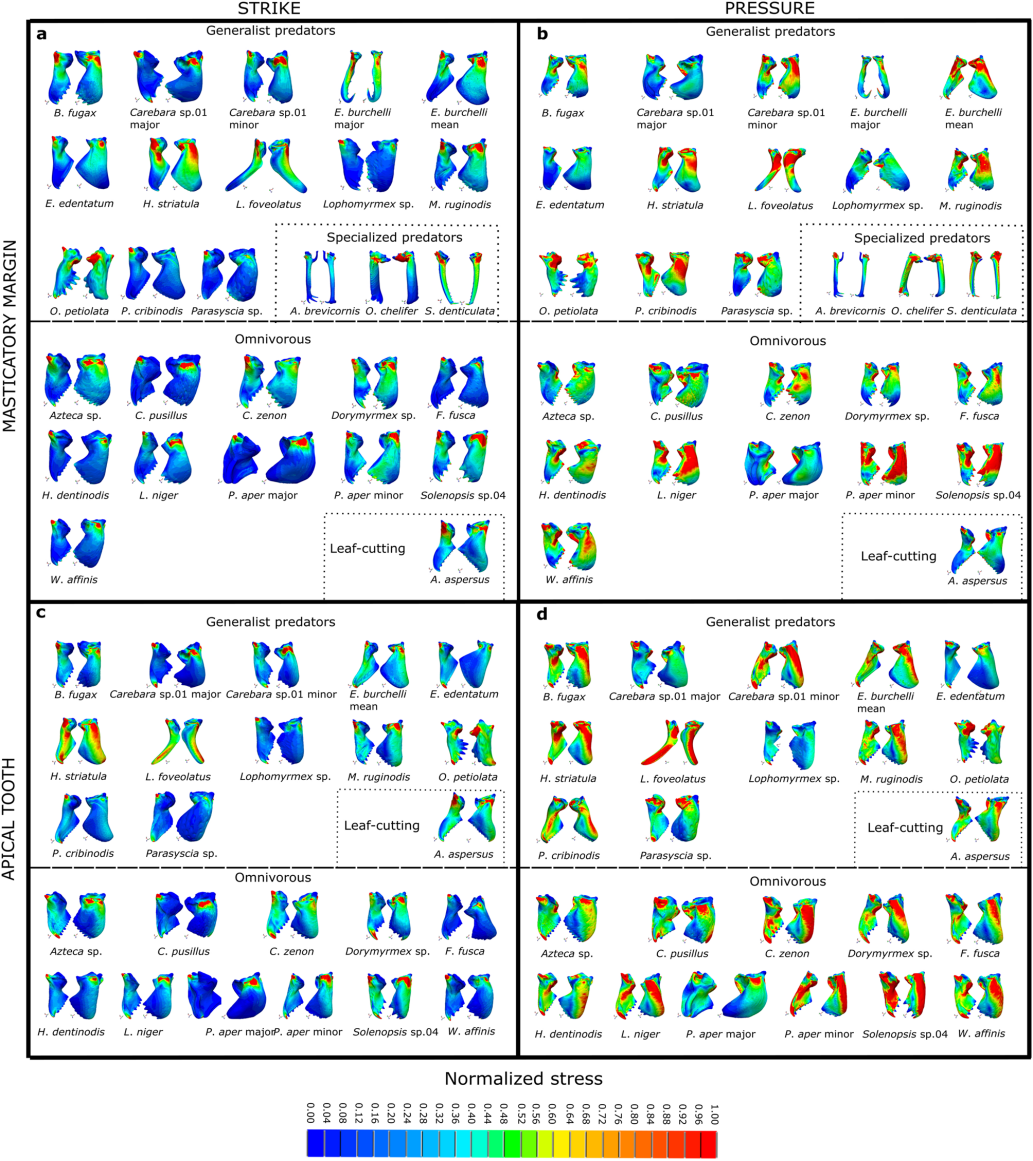
Colour maps of FEA from all biting scenarios simulated. Species are grouped according to their main feeding habit. Values of von Mises stress were normalized based on a reference model to allow direct comparison between species and biting scenarios. Strike with the entire masticatory margin (**a**); Pressure with the entire masticatory margin; (**c**) Strike with the apical tooth; (**d**) Pressure with the apical tooth.

In strike simulations, most mandibles of predatory and omnivorous species concentrate relatively higher stresses around the mandibular articulations with the head, while the stresses were comparatively lower along the mandible blade. Some species, however, showed a large surface area with intermediate to high stress levels along the mandible blade. Among predatory species, *Acantognathus brevicornis*, the major worker of *Carebara* sp.01, *Lophomyrmex* sp.01, *Platythyrea cribrinodis*, and *Parasyscia* sp. had high-stress concentrations around the mandibular articulations, whereas *Bothroponera fugax*, the minor worker of *Carebara* sp.01, the media worker of *Eciton burchellii*, *Ectatomma edentatum*, *Myrmica ruginodis*, and *Octostruma petiolata* also showed high levels of stress along the mandible blade. Similarly, omnivorous species such as *Cephalotes pusillus*, *Formica fusca*, *Heteroponera dentinodis*, *Lasius niger*, *Odontomachus chelifer*, the major worker of *Pheidole aper*, and *Wasmannia affinis* concentrated high levels of stress around the mandible articulations with the head, while *Azteca* sp., *Camponotus zenon*, *Dorymyrmex* sp., the minor worker of *Pheidole aper* and *Solenopsis* sp.04 exhibited relatively high stresses along the mandible blade too. Finally, the leaf-cutting species *Acromyrmex aspersus* showed intermediate to high stress levels along the mandible blade (Fig. 1a).

In terms of pressure biting, several predator species, including the major worker of *Carebara* sp.01, *Ectatomma edentatum*, *Lophomyrmex* sp.01, *Octostruma petiolata*, and *Parasyscia* sp., showed proportionally lower levels of stress along the mandible blade, a pattern found only in the major worker of *Pheidole aper* among omnivorous species (Fig. 1b). Meanwhile, some omnivorous species, such as *Solenopsis* sp.04 and the minor worker of *Pheidole aper* showed a large surface area in their mandible blade with proportionally high stress levels (Fig. 1b,d). The leaf-cutting ant *Acromyrmex aspersus* was among the species with lower relative stress levels along the mandible blade. Although most species showed relatively higher stress levels covering a larger surface area in pressure than strike biting, there were some important exceptions to this pattern, such as the mandibles of *Acromyrmex aspersus*, *Ectatomma edentatum*, *Octostruma petiolata*, *Holcoponera striatula*, and the major worker of *Eciton burchellii*, which exhibited only subtle differences in stress distribution between biting conditions (Fig. 1).

For simulations using only the apical tooth, we excluded the mandibles of *Acantognathus brevicornis*, *Eciton burchellii*, *Odontomachus chelifer*, and *Strumigenys denticulata* due to their specialized morphologies, in which the independent use of the apical tooth is improbable. In general, when comparing the use of the entire masticatory margin to employing only the apical tooth in strike (Fig. 1a,c) and pressure (Fig. 1b,d) simulations, we found no relevant differences. However, *Holcoponera striatula* and *Lenomyrmex foveolatus* represented two exceptions, where using the apical tooth only resulted in a more spread-out distribution of higher stress levels, representing a worse stress pattern (Fig. 1c,d).

*Ectatomma edentatum* and *Holcoponera striatula*, which are closely related and exhibit similarities in their mandible morphologies, showed clear distinctions in stress distribution in strike and pressure biting. Specifically, *Holcoponera striatula* showed a larger surface area of the mandible with relatively higher stresses than *Ectatomma edentatum* (Fig. 1). This finding suggests that *Ectatomma edentatum* exhibits superior biting performance compared to *Holcoponera striatula*.

### Intervals method

**Figure 2:**
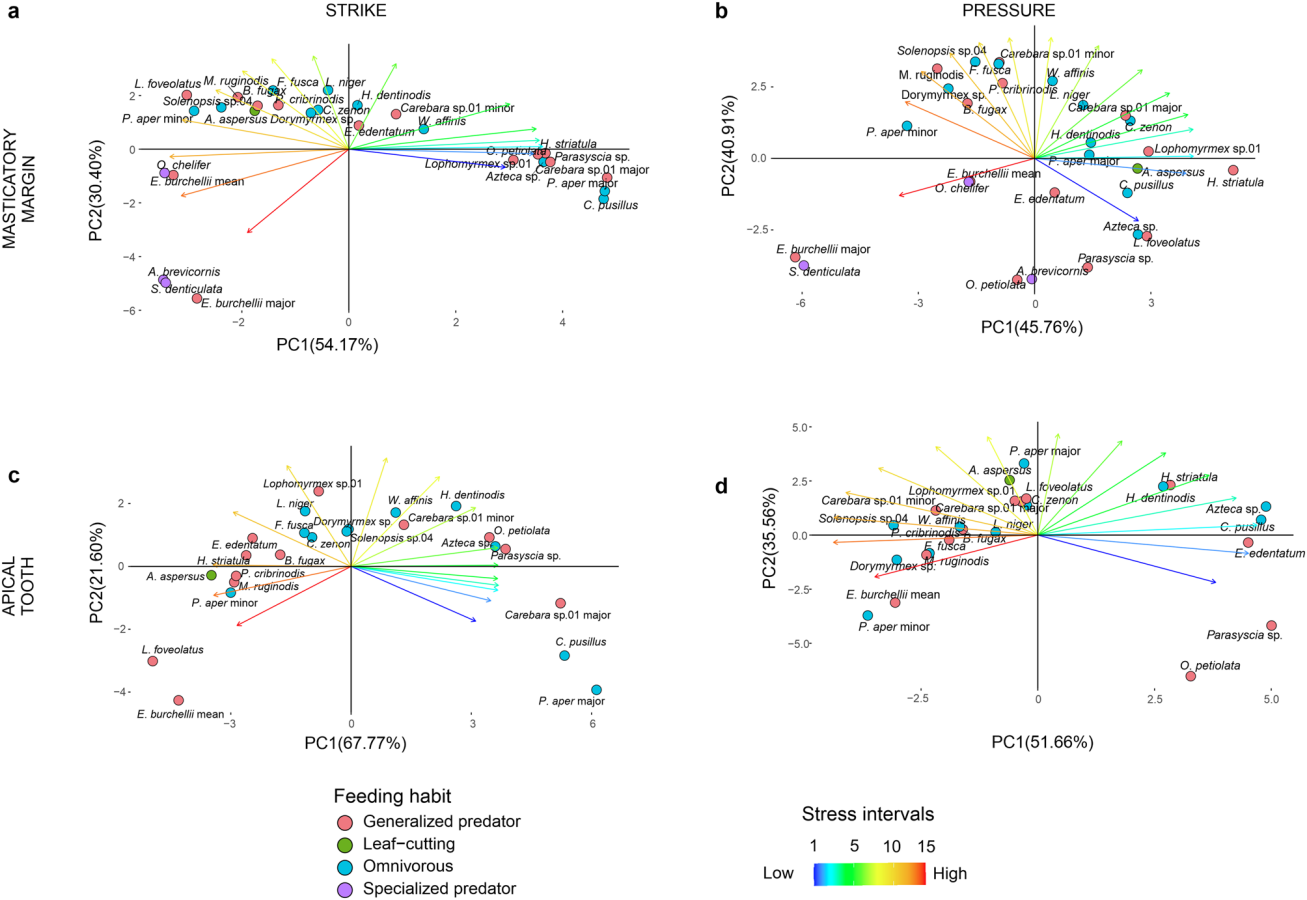
PCA based on the proportion of mandibular volume filled with each of the 15 stress intervals for all bite scenarios simulated: strike (**a**) and pressure (**b**) with the entire masticatory margin, strike (**c**) and pressure (**d**) with the apical tooth only. Colored circles depict the species main feeding habit, whereas colored arrows depict stress intervals, from the lowest stress value (blue—1) toward the highest (red—15).

To further explore the interspecific differences in mandibular stress distribution, we conducted PCAs for each biting scenario using the percentage of mandibular volume covered by 15 stress intervals as input variables. In strike simulations with the entire masticatory margin, the first two components of the PCA explained 85% of the variance. PC1, which accounted for 54% of the variance, represented stress intervals ranging from low to high, except for the highest stress interval, which was more closely associated with PC2. An increase in PC1 indicated essentially a larger mandibular volume covered by intervals of low stress (Fig. 2a). The negative range of PC1 included *Eciton burchellii* workers, *Odontomachus chelifer*, *Strumigenys denticulata* and *Acantognathus brevicornis*, all of which had a larger mandibular volume filled with higher stress intervals. In contrast, the positive range of PC1 featured species such as *Cephalotes pusillus* and the major workers of *Carebara* sp.01 and *Pheidole aper*, which had a larger mandibular volume filled with the lowest stress intervals (Fig. 2a). PC2 explained 30% of the variance and mainly distinguished mandibles with a higher coverage of the highest stress levels (interval 15) from the remaining species in its negative range. This pattern was observed in the major worker of *Eciton burchellii*, *Strumigenys denticulata*, and *Acantognathus brevicornis*. The positive range of PC2 was associated with an increase of intervals 5–9, but no species or group of species was distinctly isolated in this region of the PCA (Fig. 2a).

For simulations of pressure with the entire masticatory margin, the first two components of the PCA accounted for 87% of the variance. PC1 explained 46% of the variance and primarily distinguished species with a proportionally larger mandibular volume displaying higher stress (such as the major worker of *Eciton burchellii* and *Strumigenys denticulata*) from species more associated with intervals 2–5 (such as *Holcoponera striatula*) (Fig. 2b). PC2 explained 41% of the variance and was positively associated with intervals 8–12. It mainly differentiated *Octostruma petiolata* and *Acantognathus brevicornis* from other species in its negative range, as these species had lower proportions of their mandibles filled with such intervals (Fig. 2b).

In strike simulations using only the apical tooth, PC1 explained 68% of the variance and showed a positive association with intervals 2–7 while being negatively associated with intervals 13–14. Along the negative range of PC1, *Lenomyrmex foveolatus* and the median worker of *Eciton burchellii* displayed a larger mandibular volume with higher stress levels. In contrast, on the PC1 positive range*, Cephalotes pusillus*, the major worker of *Carebara* sp.01 and *Pheidole aper* stood out for having a larger mandibular volume with the lowest stress interval (Fig. 2c). PC2 explained 22% of the variance and was associated with a larger mandibular volume filled with intervals 9–11. This axis mainly differentiated the major worker of *Pheidole aper* and the median worker of *Eciton burchellii* from other species in its negative range, given their lower proportion of mandibular volume filled with such stress intervals (Fig. 2c).

In simulations of pressure using only the apical tooth, the first two components accounted for 87% of the variance. PC1 explained 52% of the variance and showed a positive association with intervals 2–4 while being negatively associated with intervals 12–14. This axis mainly differentiated *Azteca* sp., *Cephalotes pusillus*, *Parasyscia* sp., and *Ectatomma edentatum* from other species in its positive range (Fig. 2d). PC2 explained 36% of the variance and was positively associated with intervals 7–10. This axis mainly isolates *Octostruma petiolata* from other species in its negative range, as it had a lower proportion of mandibular volume covered by such intervals (Fig. 2d).

## Discussion

In this study, we aimed to investigate how mandible morphological variation influences stress patterns during biting and whether predatory ants exhibit enhanced biting performance compared to omnivorous species. We simulated the mechanical behavior of mandibles from 25 ant species belonging to different feeding habits, including predatory, omnivorous, and leaf-cutting species, and tested four biting scenarios. Bite simulations revealed no apparent specialization in biting performance between predatory and omnivorous species, and differences in stress patterns are more pronounced at the interspecific level than between feeding habits. Similar stress patterns were observed among species of all feeding habits, contrary to our initial expectation that predatory ants would exhibit superior performance compared to omnivorous species. This general pattern was reflected in the distribution of stress intervals along the mandibular volume, where groups of predatory and omnivorous species were not observed on the PCAs of all biting scenarios. The only exception to this general pattern was observed in the specialized predators (trap-jaw ants), which were generally isolated from other species on the PCAs. These ants typically had a larger volume of their mandibles filled with intervals of intermediate to high stresses, contradicting our hypothesis that trap-jaw ants would show a better mechanical performance in strike biting. The more robust mandibles of *Pheidole aper* and *Carebara* sp.01 majors showed relatively lower stresses and were associated with the lowest stress intervals in all biting conditions, partially differing from our hypothesis that such mandibles would perform better only in pressure biting.

Although there were no evident differences in stress patterns when comparing the differential use of the masticatory margin (entire or apical tooth only), we observed differences between pressure and strike biting. Pressure biting tended to generate higher relative stress levels along a larger mandibular surface area than strike bite, suggesting that pressing with mandibles is more mechanically demanding than striking. However, pressure biting tends to generate lower values of non-normalized stress than striking bite. Therefore, the mechanical demands of pressure biting are more relevant in long-lasting biting, where tiny failures can accumulate along the cuticle than in an acute high-stress peak, which is characteristic of strike biting. In some species, such as *Acromyrmex aspersus*, *Ectatomma edentatum*, *Octostruma petiolata*, *Holcoponera striatula*, and the major worker of *Eciton burchellii*, there were only subtle differences in stress distribution between pressure and strike biting, indicating a lack of specialization towards a specific biting behavior.

The form-function relationship of mandibles has been explored with FEA in other insect groups, such as Odonata^8^ and beetles^47–50,60^. Regarding ants, the relationship between mandible morphology and feeding habit is supported by data from fossil^40,61^ and current lineages^34,35,62,63^. Studies focused on ant functional groups have found relationships between mandible morphology and species feeding habits^64–66^. However, not all studies have found clear correlations. A recent study using a geometric morphometric approach with 3D data of the diverse ant genus *Pheidole* did not find significant relationships between the mandible and head shape with feeding and nesting habits^31^. A phylogenetically broader assessment suggested that predatory ants tend to have longer and straighter mandibles, with predatory and omnivorous species differing in general morphological traits, although this effect was not statistically significant^30^. Also, the mandible length of more than 400 ant species showed no correlation with trophic position^67^. Despite such reports, studies investigating the effects of mandible morphology on biting behavior in ants are still limited. Although there is evidence that mandible shape has an impact on the responses of these structures to bite-loading demands, these studies have often relied on small sets of species^26,33,38^. In this context, our study stands out by comparing a higher number of species, and indicate that the main feeding habits, such as omnivory versus predation, are not good predictors of stress patterns in ant worker mandibles when considering only the variation in mandible shape and thickness.

Among the species studied, those with stouter mandibles, such as *Cephalotes pusillus* and majors of *Carebara* sp.01 and *Pheidole aper*, are among the ones exhibiting relatively lower stress levels in pressure biting, as predicted. Interestingly, however, these stouter mandibles also showed relatively lower stresses in strike biting, as confirmed when considering normalized and non-normalized stress levels. Those species had mandibles with a broader blade and a subtle constriction near the mandibular base, a more convex curvature on the mandibular external face, and either short teeth or the absence of such structures. The lack of teeth means that the stresses generated in the masticatory margin do not concentrate heavily on smaller regions, as when teeth are present, but spread more evenly along the masticatory margin. A prominent mandibular curvature hampers the stress dissipation towards the delicate mandible blade, generating stress concentrations on the more robust masticatory margin and mandible base. *Pheidole* major workers are task specialists, often recruited for food processing (e.g. crushing seeds), resource retrieval (e.g. chopping and carrying pieces of large dead arthropods), and colony defense^68–70^, behaviors that require strong bite forces and generate higher mechanical demands than other activities. *Pheidole aper* is not a granivorous species^71^, but there seems to be no strong morphological differences between granivorous and non-granivorous species^31^. A better biting performance of *Pheidole* major worker mandibles was previously highlighted^72^, mainly in pressure biting^33^, but not in comparison with other ant genera. Majors of *Carebara* spp. perform similar specialized tasks as those described for *Pheidole*^73,74^, and their mandibular mechanical responses to bite-loading demands were correspondingly similar. Finally, species of *Cephalotes* spp. are known by their thicker exoskeleton, culminating in the morphological specialization of major heads to protect nest entrances^75,76^, but the mechanical demands of bite in worker mandibles were never tested before.

Two closely related species, *Ectatomma edentatum* and *Holcoponera striatula*, have similar mandibular morphology but show contrasting stress patterns in all biting scenarios tested, with *Holcoponera striatula* exhibiting relatively higher stress levels than *Ectatomma edentatum*. Regarding their positioning in the PCAs, both mandibles are more separated along the PC1 in simulations with the masticatory margin, suggesting that this mandibular region could represent an important source of mechanical distinction between the species. In fact, the masticatory margin in *Ectatomma edentatum* is wider (~ 0.9 mm) than in *Holcoponera striatula* (~ 0.3 mm), resulting in the bite load being applied along a broader area and hence dissipating over a larger area. However, a deeper investigation is needed to unveil the possible morphological aspects that explain the observed disparity in mechanical performance.

The trap-jaw ants, including *Acantognathus brevicornis*, *Odontomachus chelifer*, and *Strumigenys denticulata*, along with the major worker of *Eciton burchellii*, possess specialized mandibles, which in general are long, slender, with well-developed teeth, although the major worker of *Eciton burchellii* differed from the trap-jaw ants in having a hook-shaped mandible with a single tooth. Despite sharing these mandibular characteristics, those four mandibles displayed distinct stress patterns. *Strumigenys denticulata* and the major worker of *Eciton burchellii* showed proportionally higher stress levels throughout the mandibles than *Acantognathus brevicornis* and *Odontomachus chelifer*. However, when considering the non-normalized stress intervals, all these mandibles were highlighted on the PCAs by showing a proportionally larger volume filled with higher stress intervals, particularly in strike biting. These results do not support the initial hypothesis that trap-jaw ants would show relatively lower stress levels in strike biting. This partial contrast between the results of color maps and stress intervals provides intriguing suggestions about how trap-jaws deal with biting mechanical demands. In general, the long and slender mandibular blade withstands much lower stresses, reducing the risk of failure, but different from what was suggested for the snap-jaw ant *Mystrium camillae*, its general morphology and cross-section geometry does not indicate that such mandibles are prone to deform and withstand stress^26^, but this requires further investigation. Evidence from *Odontomachus monticola* highlights the importance of mandibular hollowness in withstanding the stresses generated by their powerful strike bites^38^. The natural hollowness of this species appears to optimize the trade-off between power generation and impact resistance, compared to mandibles with higher indices of hollowness or completely solid mandibles^38^. Accordingly, for all species tested in our study, their natural hollowness and cuticle thickness was kept for the simulations.

Although this study has shown some effects of mandible shape variation on stress distribution, it is important to note that the approach here presented does not fully capture the complexity of the mandible cuticle’s material properties. Insects have a gradient of material properties along the layers of their cuticle^77^, which varies across different body regions. This material gradient proved to be functionally significant in some studies^78–80^ and is particularly relevant to ant mandibles, which can accumulate heavy metals along their masticatory margin, leading to increased cuticular stiffness in those regions^81–85^. However, information about the variation in material properties of ant mandibles is still limited, with only a few studies restricted to a few leaf-cutting ants^81,86^. Moreover, incorporating material property variation in FEA increases the simulation complexity and can lead to confounding effects on structure shape and material properties^56^. Therefore, it is essential to consider the influence of material property variation under specific hypotheses and with appropriate data on the species in question. Further efforts are needed to measure the heterogeneity of cuticle material properties in ant mandibles to provide a better picture of their mechanical behavior.

Biomechanical approaches such as FEA are interesting tools for testing hypotheses about the role of mandible morphology in bite mechanics. Our results suggest that more robust mandibles exhibited lower stress levels under both pressure and strike biting, whereas mandibles of trap-jaw ants did not show signs of adaptation to strike biting in relation to the remaining species. Also, the main feeding habits of the ant species tested do not anticipate the general stress patterns observed in their mandibles under bite loading, being that the interspecific morphological disparity seems more relevant to biting performance among the ants tested. Ant mandibles are essential for food capture and processing but also play a crucial role in other colony tasks performed by workers^10,54^. Our effort builds on previous studies that found weak associations between mandible morphology and feeding ecology in ants^30,31^. Therefore, mandible morphology could reflect the necessity to perform multiple tasks^8,54^ or to excel in other activities, such as excavation^32^, hypotheses that demand further studies to be properly tested.

The evolution of ant mandibles has been the subject of intense study in recent years^18,28,29,41^, and although the shovel-shaped mandible is considered the Bauplan of extant ant mandibles^29^, many ant lineages exhibit significant deviations from this general morphology (see also^62^). Our sampling set covers a significant sample of this variation and relevant functional differences were observed. Further studies may explore the effects of variation in cuticle material properties, particularly considering the interspecific variation in heavy metal accumulation^85^. It would also be meaningful to investigate more widely the relationship between mandible morphology and bite force in ants^87,88^.

## Methodology

### Species selection

To simulate biting behavior, we selected one worker specimen of 25 ant species with a wide range of mandible morphologies, which were representative of two primary feeding habits: predatory (14 species) and omnivorous (10), along with a leaf-cutter ant (Table 1). For a few polymorphic ant species, we also considered a second worker type (Table 1). To establish the general feeding habits of these species, we collected information from online repositories (e.g.^89^) and literature sources^90,91^. In cases where we did not find the species’ main feeding habit or were able to identify the species only at the genus level (morphospecies), we considered the main feeding habit of the genus. We classify as predators the ant species known to actively hunt other animals (mainly arthropods) because of the mechanical demands of subduing a prey, which is assumed to differ from simply feeding on dead animals. For the predatory category, we included species with specialized mandible morphology and mechanics, the trap-jaw ants. Due to their specialized mandibles, these species were classified here as specialized predators, although not necessarily feeding exclusively on live prey. Omnivorous species do not actively predate other organisms as their primary feeding habit, usually relying on many different food sources without subduing living prey. We also added a leaf-cutting ant (*Acromyrmex aspersus*), which exhibits a unique biting behavior for cutting leaves. We aimed to broadly categorize species based on their primary differences in feeding mechanical demand, not to describe in detail their natural diets, since many species are known to opportunistically feed on a wide range of trophic sources^71^. In cases where different species displayed similar mandible morphology, we relied on the quality of the µCT scans (see below) to decide which species would enter our final dataset. Species identifications were confirmed with assistance from taxonomists (see Acknowledgments). All scanned specimens are deposited in the Formicidae collection of the “Coleção Entomológica Mítia Heusi Silveira”, Universidade Federal de Santa Catarina, Brazil (Supplement S1).

**Table 1:** List of species considered for finite element analysis in this study. Also shown are the worker type, energy and voxel size of the scans, surface area of the meshes (considered for load normalization), number of elements of each mesh, the applied load and the feeding habit of the species.

Species	Worker type	Energy (keV)	Voxel size (µm)	Surface area (mm^2^)	Number of elements	Applied load (N)	Feeding habit
*Acanthognathus brevicornis* Smith, 1944	Normal	11	0.64	0.116002	1,306,962	0.0343	Specialized predator
*Acromyrmex aspersus* (Smith, 1858)	Normal	12	2.09	0.777902	645,215	0.2300	Leaf-cutting
*Azteca* sp.	Normal	11	1.28	0.161742	545,853	0.0478	Omnivorous
*Bothroponera fugax* (Forel, 1907)	Normal	10	1.25	0.265897	361,389	0.0786	Generalized predator
*Camponotus zenon* Forel, 1912	Normal	12	2.45	0.463858	656,142	0.1372	Omnivorous
*Carebara* sp.01	Major	20	2.44	1.191900	919,446	0.3524	Generalized predator
	Normal	20	1.22	0.130098	445,297	0.0385	
*Cephalotes pusillus* (Klug, 1824)	Normal	11	0.64	0.090715	428,956	0.0268	Omnivorous
*Dorymyrmex* sp.	Normal	11	0.64	0.056881	501,913	0.0168	Omnivorous
*Eciton burchellii* (Westwood, 1842)	Major	11	2.40	3.382100	1,012,354	1.0000	Generalized predator
	Media	10	1.25	0.444433	755,864	0.1314	
*Ectatomma edentatum* Roger, 1863	Normal	20	3.67	2’.132400	886,718	0.6305	Generalized predator
*Formica fusca* Linnaeus, 1758	Normal	12	2.09	0.466144	876,613	0.1378	Omnivorous
*Heteroponera dentinodis* (Mayr, 1887)	Normal	12	2.09	0.244014	781,636	0.0721	Omnivorous
*Holcoponera striatula* (Mayr, 1884)	Normal	12	2.09	0.300672	175,223	0.0889	Generalized predator
*Lasius niger* (Linnaeus, 1758)	Normal	12	2.09	0.234028	448,285	0.0692	Omnivorous
*Lenomyrmex foveolatus* Fernández & Palacio, 1999	Normal	11	0.64	0.066555	255,517	0.0197	Generalized predator
*Lophomyrmex* sp.01	Normal	20	2.44	0.254868	1,153,231	0.0754	Generalized predator
*Myrmica ruginodis* Nylander, 1846	Normal	12	1.31	0.142068	915,314	0.0420	Generalized predator
*Octostruma petiolata* (Mayr, 1887)	Normal	12	2.09	0.100413	342,483	0.0297	Generalized predator
*Odontomachus chelifer* (Latreille, 1802)	Normal	20	3.67	2.821800	1,123,856	0.8343	Specialized predator
*Parasyscia* sp.	Normal	11	1.2	0.149357	618,498	0.0442	Generalized predator
*Pheidole aper* Forel, 1912	Major	12	1.31	0.409701	861,502	0.1211	Omnivorous
	Normal	12	0.66	0.090171	364,652	0.0267	
*Platythyrea cribrinodis* (Gerstäcker, 1859)	Normal	11	2.40	1.269600	664,280	0.3754	Generalized predator
*Solenopsis* sp.04	Normal	11	1.2	0.082008	282,457	0.0242	Omnivorous
*Strumigenys denticulata* Mayr, 1887	Normal	12	1.22	0.086116	581,390	0.0255	Specialized predator
*Wasmannia affinis* Santschi, 1929	Normal	12	0.66	0.024214	608,544	0.0072	Omnivorous

### Scanning and reconstruction of volumetric models

Ant specimens were scanned using synchrotron radiation X-ray tomography at two German facilities, Imaging Beamline P05 (IBL)^92–94^ operated by the Helmholtz-Zentrum-Geesthacht at the storage ring PETRA III (Deutsches Elektronen Synchrotron—DESY, Hamburg, Germany)—and KIT Light Source (Eggenstein-Leopoldshafen). As a result of the tomographic reconstruction the scans were assembled as Tiff image series for subsequent segmentation, with scanning parameters varying depending on the ant species (Table 1). Tomographic reconstruction for P05 data has been done using a custom reconstruction pipeline^95^ using Matlab (Math-Works) and the Astra Toolbox^96–98^. Volumetric CT data of ant worker mandibles were then pre-segmented using the software *Amira 5.4* (Visage Imaging GmbH, Berlin, Germany). A set of slices was manually segmented using the *Magic Wand* tool, at intervals ranging from 5 to 15 slices, depending on the complexity of the mandible and the quality of the scans. Automatic interpolation between the pre-segmented slices was done using the online platform *Biomedisa*^99^. To ensure that the reconstructions accurately represented the morphology of ant mandibles, the outputs from *Biomedisa* were imported back into *Amira 5.4*, where we corrected inaccuracies and reduced the complexity of the reconstructed morphology, taking into account the presence of hairs, sharp edges, and holes, among other factors. Cuticle thickness can vary substantially along the ant mandible, and the degree of mandibular hollowness can also differ between species, both being potentially relevant to its bite mechanics^38^. As such, the natural hollowness and cuticle thickness of each mandible were represented in our mandible digital representations.

### Finite element mesh generation

The mandible surface models were imported into *Blender 2.93* (https://www.blender.org/) to position them in a common orientation. We then used *FUSION 360* (AUTODESK) to decrease mesh density. By conducting mesh convergence simulations, we determined a minimum mesh density for each mandible that approximates the 3D mandible morphology and provides a lower computational demand to solve the finite element equations^100^. To emulate actual simulation parameters, we generated a simplified version of the final simulation setup, sampling three to six elements from specific mandibular regions to check for convergence (less than 5% of error) on von Mises stress values^101^. We exported meshes from FUSION 360 as *.stl* files and imported them into FEBio^102^, where we conducted convergence tests and the final FEA simulations. The number of elements for the definitive meshes is available in Table 1. Volumetric representations of each worker mandible are available as supplementary material (File S2).

### Finite element analysis

**Figure 3:**
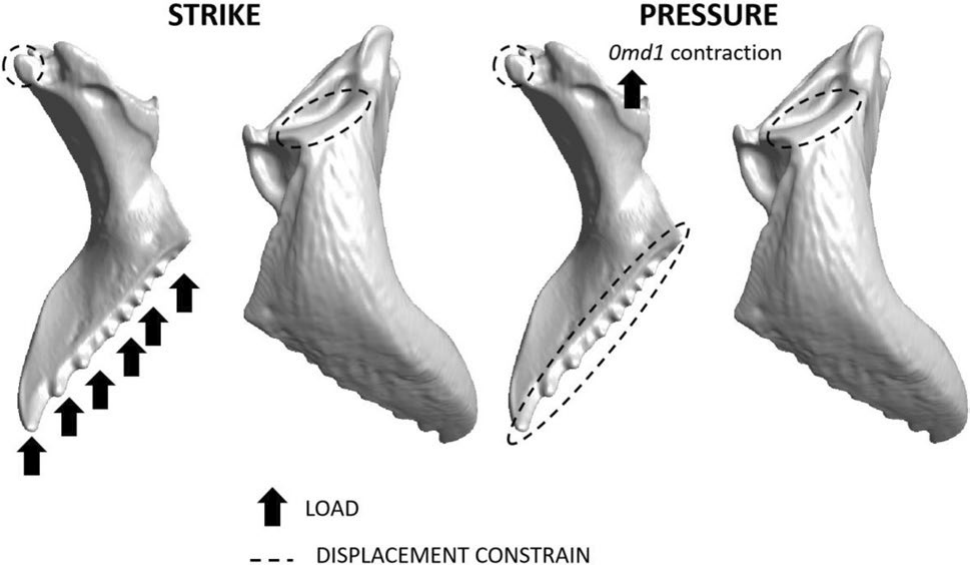
Diagrams depicting the boundary conditions for each biting scenario. Highlighted are the conditions for strike and pressure biting with the entire masticatory margin.

In this study, we aimed to investigate the effect of mandible morphology on bite-loading demands by simulating four bite conditions that reflect various phases of biting and the employment of different parts of the masticatory margin. Specifically, we simulated mandible use in strike and pressure biting using the entire masticatory margin and employing only the apical tooth. Strike biting emulates the usage of mandibles to hit an object and represents the first phase of bite. To simulate strike biting, we applied a load on the masticatory margin or the apical tooth of the mandible, restricting the nodal displacement on the mandibular articulations with the head in all directions (Fig. 3). Similarly, for the second bite phase, which we refer to as pressure biting and which involves crushing or gripping an object, we applied a load on the mandibular region where the mandibular apodeme inserts, following the direction of forces generated by *0md1* contraction. We also restricted the mandible articulations with the head and the masticatory margin or apical tooth to zero nodal displacements in all directions. To ensure consistency, we applied the same material properties to each simulation. The Young's modulus was defined as 2.75 GPa based on measurements from the mandibles of *Atta laevigata* available in the literature^86^, and the Poisson ratio was set at 0.3, as commonly considered for ant cuticle^26,33,38,54^. To account for differences in mandible size, we applied a 1 N load to the largest mandible and adjusted the load for the remaining mandibles based on surface area differences^103^ (Table 1). Restricting parameter variation to mandible morphology alone, such as mandible shape and cuticle thickness, allows us to compare results between simulations^56^. This approach is commonly used in comparative studies^8,26,33,48–50^.

### Intervals method

The Intervals Method is an approach developed to quantify the area (2D) or volume (3D) of structures covered by a specific range of stress^104^. This method involves using information about element stress and volume to define intervals of stress values and calculate the proportion of the structure volume filled with each stress interval. An upper threshold of stress value must be defined, which is a reference for the determination of the remaining stress intervals. It is possible to define any number of stress intervals from this threshold value and to compute the volume occupied by those intervals on each model. The amount of the structure volume filled with each interval represents new variables that can be used in further statistical tests or ordination analyses, such as principal component analyses (PCA)^104^. This approach allows for a direct comparison of stress distribution between the mandibles in a more quantitative way than the color maps, where normalized stress values are being compared. In comparing the proportion of mandibular volume filled by each stress interval, we can observe which structures are submitted to higher or lower non-normalized stresses, and how relevant those stresses are for the structure as a whole, which is informative when a size-corrected load is considered for the simulations, as was done here.

We applied the method independently to each biting scenario. We extracted data on von Mises stress and volume from the elements of each simulation from FEBio^102^. Then we removed elements that corresponded to the 2% higher stress values in each simulation, as these values often represent artificially high-stress values^104,105^. Even after the removal of such potentially artificial high-stressed elements, a few mandibles showed substantially higher stress values than the remaining ones, achieving values up to one order of magnitude higher (Fig. S1). To account for this variation in the stress scale and avoid eventual biases toward the highly stressed mandibles (Fig. S1), we log-transformed stress values prior to generating the stress intervals. We defined the upper threshold values for each biting scenario to include the 15% highest stress values above the threshold. Defining a higher threshold value could lead to uninformative intervals, as many models would have no elements within the highest interval due to the variation in the range of stress generated among models (Fig. S1). To define the ideal number of stress intervals, we generated datasets with different numbers of intervals (5, 10, 15, 25, 50) and performed PCAs. We considered PC1 and PC2 scores of each dataset in linear regressions with the scores of equivalent PCs of the next interval *(*e.g., PC1_5_intervals_~PC1_10_intervals_), and we retrieved the coefficient of determination (R^2^) to analyze the convergence of PC scores. The stop of increase in R^2^ defined the final number of intervals^104^. For all biting scenarios, convergence occurred with 15 intervals, so we used this number for the PCAs (File S3). We conducted our analyses in R version 4.1.3^106^ using the *FactoMineR* version 2.4^107^ and *factoextra* version 1.0.7.999^108^ packages.

To summarize, we provide our FEA results in two ways: color maps and the intervals method^104^. Color maps were graphical outputs generated by FEA software displaying stress distribution along the mandibles. To compare results between species and biting scenario stress values were normalized using a reference model. The resulting color variation on the maps represents the proportion of stress based on the maximum normalized stress value for each simulation (Fig. 1). In contrast, the intervals method involved quantifying the volume of the mandible that falls within specific stress intervals for each of the four biting scenarios^104^. These proportions served as input variables for the PCAs used to address differences in stress distribution among ant mandibles across the 15 stress intervals (Fig. 2).

### Supplementary Information

Supplementary Figure S1.

Supplementary Figure S2.

Supplementary Figure S3.

Supplementary Information 4.

## Data Availability

Collection information of the sampled ant species (Supplement S1), volumetric meshes of the mandibles considered for FEA (Supplement S2), as well as the intervals method data for each biting scenario (Supplement S3) are available as supplementary files. R code regarding the application of the intervals method is available in the Supplementary Material accompanying this article.
