# Supplementary material for: Simulated biomechanical performance of morphologically disparate ant mandibles under bite loading
Source: Sci Rep. 2023 Oct 6;13:16833. doi: 10.1038/s41598-023-43944-8 (PMC10558566; doi:10.1038/s41598-023-43944-8)
Supplement: Supplementary file 4 — Supplementary Information 4. [file 41598_2023_43944_MOESM4_ESM.pdf]

SUPPLEMENTARY MATERIAL

Simulated biomechanical performance of morphologically disparate ant mandibles under bite loading

Klunk, C.L.[1,2*]; Argenta, M.A.[3]; Rosumek, F.B.[4]; Schmelzle, S.[2]; van de Kamp, T.[5,6]; Hammel, J.U.[7]; Pie, M.R.[8]; Heethoff, M.[2*]

[1]Graduate Program in Ecology and Conservation, Universidade Federal do Paraná, Curitiba, PR, Brazil.

[2]Animal Evolutionary Ecology, Technische Universität Darmstadt, Schnittspahnstr. 3, D-64287, Darmstadt, Germany.

[3]Department of Civil Construction, Universidade Federal do Paraná, Curitiba, PR, Brazil.

[4]Department of Ecology and Zoology, Universidade Federal de Santa Catarina, Florianópolis, SC, Brazil.

[5]Institute for Photon Science and Synchrotron Radiation (IPS), Karlsruhe Institute of Technology (KIT), Eggenstein-Leopoldshafen, Germany.

[6]Laboratory for Applications of Synchrotron Radiation (LAS), Karlsruhe Institute of Technology (KIT), Karlsruhe, Germany.

[7]Institute of Materials Physics, Helmholtz-Zentrum Hereon, Geesthacht, Germany.

[8]Biology Department, Edge Hill University, Ormskirk, Lancashire, United Kingdom.

[*]klunkcristian@gmail.com; heethoff@bio.tu-darmstadt.de

File S1. Geographic and identification information of each species considered for the FEA simulations.

File S2. Volumetric meshes of ant worker mandibles used for biting simulations.

File S3. Proportion of mandibular volume filled by each stress interval for each biting scenario, used for the generation of the PCAs depicted in Fig. 2 of the main text.

matrix-of-15-intervals_pressure_AT_logNN: Proportion of mandibular volume filled by each stress interval regarding pressure with the apical tooth only. Stress data was log-corrected before the generation of stress intervals. Species are represented by the rows, the 15 stress intervals are depicted in the columns.

matrix-of-15-intervals_strike_AT_logNN: Proportion of mandibular volume filled by each stress interval regarding strike with the apical tooth only. Stress data was log-corrected before the generation of stress intervals. Species are represented by the rows, the 15 stress intervals are depicted in the columns.

matrix-of-15-intervals_pressure_MM_logNN: Proportion of mandibular volume filled by each stress interval regarding pressure with the entire masticatory margin. Stress data was log-corrected before the generation of stress intervals. Species are represented by the rows, the 15 stress intervals are depicted in the columns.

matrix-of-15-intervals_strike_MM_logNN: Proportion of mandibular volume filled by each stress interval regarding strike with the entire masticatory margin. Stress data was log-corrected before the generation of stress intervals. Species are represented by the rows, the 15 stress intervals are depicted in the columns.

Supplementary figures.

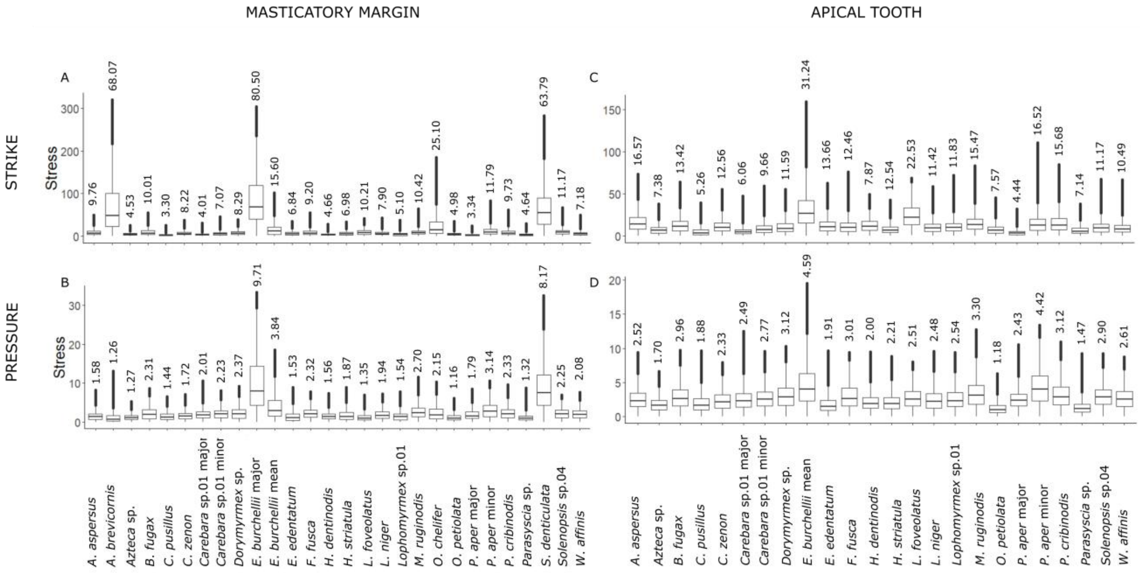

Fig.S1 Boxplots depicting the distribution of non-normalized stress of each species for each biting behavior, based on the Tresca failure criteria, after removing the 2% higher stress value for each simulation. Boxes represent the interquartile range (IQR), with its limits depicting the 25th (Q1 - lower) and 75th (Q3 - upper) percentile, and the transverse bar represents the median value of stress. Vertical lines departing from the main box represent the minimum (Q1 - 1.5 * IQR) and maximum (Q3 + 1.5 * IQR) stress values. Dots represent potential outliers. Numbers above the potential outliers represent the Mesh-Weighted Arithmetic Mean (MWAM), which are the mean stress value for each simulation corrected by the effect of element size (Marcé-Nogué et al. 2016).

REFERENCES

Marcé-Nogué, J., De Esteban-Trivigno, S., Escrig, C. & Gil, L. Accounting for differences in element size and homogeneity when comparing Finite Element models: Armadillos as a case study. *Palaeontologia Electronica* (2016).

R code for the application of the intervals method. Adapted from: Marcé-Nogué, J., De Esteban-Trivigno, S., Püschel, T. A. & Fortuny, J. The intervals method: a new approach to analyse finite element outputs using multivariate statistics. PeerJ 5, e3793 (2017)

```
setwd("")

library(dplyr)
library(ggplot2)
library(viridis)
library(tidyverse)
library(hrbrthemes)
library(stringr)
library(FactoMineR)
library(factoextra)
library(ggpubr)

#One species loading example:

A.aspersus_strikeMM <- read.table("A.aspersus_strike_mm.txt", header = T, sep = ",")
A.aspersus_strikeMM$mises <- A.aspersus_strikeMM$mises*100000000#set the correct scale of stress #(nN/µm²)

A.aspersus_strikeMM_volume <- read.table("A.aspersus_strike_mm_volume.txt", header = T, sep = ",")
A.aspersus_strikeMM_volume$volume <- A.aspersus_strikeMM_volume$volume/1000000000000#set #the correct scale of volume (µm³)

#complementing the dataframe (not all identifiers will be used along the remaining of the code)

A.aspersus_strikeMM$species <- "A.aspersus"
A.aspersus_strikeMM$simulation <- "strike"
A.aspersus_strikeMM$diet <- "leaf-cutting"
A.aspersus_strikeMM$volume <- A.aspersus_strikeMM_volume$volume

species_results <- rbind(A.aspersus_strikeMM, A.aspersus_pressureMM, A.brevicornis_strikeMM, A.brevicornis_pressureMM, Azteca_sp_strikeMM, Azteca_sp_pressureMM, B.fugax_strikeMM, B.fugax_pressureMM, C.zenon_strikeMM, C.zenon_pressureMM, Carebara_minor_strikeMM, Carebara_minor_pressureMM, Carebara_major_strikeMM, Carebara_major_pressureMM, C.pusillus_strikeMM, C.pusillus_pressureMM, Dorymyrmex_sp_strikeMM, Dorymyrmex_sp_pressureMM, E.burchelli_major_strikeMM, E.burchelli_major_pressureMM, E.burchelli_mean_strikeMM, E.burchelli_mean_pressureMM, E.edentatum_strikeMM, E.edentatum_pressureMM, F.fusca_strikeMM, F.fusca_pressureMM, H.striatula_strikeMM, H.striatula_pressureMM, H.dentinodis_strikeMM, H.dentinodis_pressureMM, L.foveolatus_strikeMM, L.foveolatus_pressureMM, L.niger_strikeMM, L.niger_pressureMM, Lophomyrmex_sp_strikeMM, Lophomyrmex_sp_pressureMM, M.ruginodis_strikeMM, M.ruginodis_pressureMM, O.chelifer_strikeMM, O.chelifer_pressureMM, O.petiolata_strikeMM, O.petiolata_pressureMM, P.aper_major_strikeMM, P.aper_major_pressureMM, P.aper_minor_strikeMM, P.aper_minor_pressureMM, P.cribinodis_strikeMM, P.cribinodis_pressureMM, Parasyscia_sp_strikeMM, Parasyscia_sp_pressureMM, S.denticulata_strikeMM, S.denticulata_pressureMM, Solenopsis_sp04_strikeMM, Solenopsis_sp04_pressureMM, W.affinis_strikeMM, W.affinis_pressureMM)#combining all data regarding simulations with the #masticatory margin into a single dataframe for further analysis
```

```
species_results%>%
  group_by(species,simulation)%>%
  slice_min(mises, prop = 0.98)->
  species_results2#removing the 2% highest stress values of each simulation

species_results2$log.stress <- log(species_results2$mises)#log-transforming the data

strike_mm <- subset(species_results2, simulation == "strike")#subset with strike simulations only
pressure_mm <- subset(species_results2, simulation == "pressure")#subset with pressure simulations
#only

species_results2$id <- str_c(species_results2$species, '_' ,species_results2$simulation)#create an unique
#id for species and simulation

species_results2 %>%
  group_by(id) %>%
  mutate(Max = max(mises)) ->
  species_results2

species_results3 <- species_results2[, -c(2:5)]#remove unnecessary columns to apply the intervals
#method

species_results3 <- species_results3[, c(1,4,2,3)]#reordering the columns to fit the configuration of the
#function to create the stress intervals (below)

#The remaining of the code deals with the generation of stress intervals and PCA. It was adapted from
#(Marcé-Nogué, J., De Esteban-Trivigno, S., Püschel, T. A. & Fortuny, J. The intervals method: a new
#approach to analyse finite element outputs using multivariate statistics. PeerJ 5, e3793 (2017))

species_results_split <- split(species_results3, species_results3$id) # list of dataframes for each species
#and simulation type

species_results_split <- lapply(species_results_split, function(x) x[!(names(x) %in% "id")])


# use numbers as file names
lapply(names(species_results_split),
       function(x){write.csv(species_results_split[[x]], paste0(x,"MM_NNlog.csv"),#NN = non-normalized
                    row.names = FALSE)})

# 1) Set the input parameters for the method (these values are defined by the user):
# Fixed Upper Threshold FTupper

FTupper = 1.495

#upper threshold for each biting scenario:
#3.45strike_MMlog15% -> strike with the entire masticatory margin, with log transformed von Mises
#stress values, with the highest stress interval containing the 15% higher-stressed elements;
#1.463pressure_MMlog15% -> pressure with the entire masticatory margin, with log transformed von
#Mises stress values, with the highest stress interval containing the 15% higher-stressed elements;
#3.075strike_ATlog15% -> strike with the apical tooth only, with log transformed von Mises stress values,
#with the highest stress interval containing the 15% higher-stressed elements;
#1.495pressure_ATlog15% ->  pressure with the apical tooth only, with log transformed von Mises stress
#values, with the highest stress interval containing the 15% higher-stressed elements.


# Number of intervals: NIntervals
```

```
NIntervals = 15

# 2) Read the data.
# The data must be stored as .csv files in the same folder of the script
# Each .csv file must contain three rows with: 1) the number of the element,
# 2) area/volume of the element and 3) von mises stress, respectively.

file.name = list.files(pattern="*_pressureMM_NNlog.csv")
NFiles = length(file.name)

# 3) Create the matrix of intervals
# Each row with the area percentage for each interval and each file of the matrix
# with the different models included

data.intervals = matrix(ncol = NIntervals, nrow = NFiles)

for (f in 1:NFiles) {

  data.values = data.matrix(read.csv(file.name[f], header = TRUE, sep = ","));

  # Get the number of mesh elements of the model

  NElements = nrow(data.values);

  # Create the internal matrix to store the intervals and other data

  Counter.matrix = matrix(0,ncol = NIntervals, nrow = 5);

  # Compute the range values for each interval (Tlower and Tupper)

  Range.values = seq(0, FTupper, by=FTupper/(NIntervals-1));

  # Start the Loop

  for (i in 1:NElements) {
    for (j in 1:NIntervals) {
      if (j == 1){
        if (data.values[i,3] <= Range.values[j+1])
        {
          Counter.matrix[2,j]=Counter.matrix[2,j]+1;
          Counter.matrix[4,j]=Counter.matrix[4,j]+data.values[i,2];
        }
      }
      else if (j > 1 & j < NIntervals){
        if (data.values[i,3] > Range.values[j] & data.values[i,3] <= Range.values[j+1])
        {
          Counter.matrix[2,j]=Counter.matrix[2,j]+1;
          Counter.matrix[4,j]=Counter.matrix[4,j]+data.values[i,2];
        }
      }
      else if (j == NIntervals){
        if (data.values[i,3] > Range.values[j])
        {
          Counter.matrix[2,j]=Counter.matrix[2,j]+1;
          Counter.matrix[4,j]=Counter.matrix[4,j]+data.values[i,2];
        }
```

```
    }
   }
  }

  # End of the loop

  # Compute the percentage in each interval with respect  to the total area

  for (i in 1:NIntervals) {
    Counter.matrix[1,i]=Range.values[i];
    Counter.matrix[3,i]=100*Counter.matrix[2,i]/NElements;
    Counter.matrix[5,i]=100*Counter.matrix[4,i]/sum(data.values[,2]);
  }

  # Store the vector of intervals for the model f in the matrix of intervals

  data.intervals[f,]=Counter.matrix[5,];

}

data.intervals=as.data.frame(data.intervals);
row.names(data.intervals)=file.name;

# 4) End of the script: save data and remove variables

write.csv(data.intervals,'matrix-of-15-intervals_pressure_MM_logNN.csv');

rm(NIntervals, FTupper, NElements, i, j, f, file.name, NFiles, Range.values, Counter.matrix, data.values);
cat("\f");
print(paste0("Matrix of intervals created and stored in a .csv file in the working dir: ", getwd()))


row.names(stress.distrib) <- c("A. aspersus", "Azteca sp.", "B. fugax", "C. pusillus",
                    "C. zenon", "Carebara sp.01 major", "Carebara sp.01 minor", "Dorymyrmex sp.",
                    "E. burchellii mean", "E. edentatum", "F. fusca",
                    "H. striatula", "H. dentinodis", "L. foveolatus", "L. niger", "Lophomyrmex sp.01",
                    "M. ruginodis", "O. petiolata", "P. aper major", "P. aper minor",
                    "P. cribrinodis", "Parasyscia sp.", "Solenopsis sp.04", "W. affinis")

# 1) Multivariate analysis PCA

stress.distrib$feeding <- c("Leaf-cutting", "Omnivorous", "Generalized predator", "Omnivorous",
                  "Omnivorous", "Generalized predator", "Generalized predator", "Omnivorous",
                  "Specialized predator", "Generalized predator", "Omnivorous", "Generalized predator",
                  "Omnivorous", "Generalized predator", "Omnivorous", "Generalized predator",
                  "Generalized predator", "Generalized predator", "Omnivorous", "Omnivorous",
                  "Generalized predator", "Generalized predator", "Omnivorous", "Omnivorous")

col.number = ncol(stress.distrib)
PCA.stress <- PCA(stress.distrib[,1:15],  graph = FALSE)

# 2) Define the parameters and create the biplot

# colors by group
group.colors = stress.distrib$feeding

#colors by variable
```

```
interval.number = nrow(PCA.stress$var$coord)
interval.vector = seq(from = 1, to = interval.number, by=1 )
interval.colors = interval.vector
interval.palette = c("blue","cyan","green","chartreuse","yellow","gold","orange","red")

# Biplot: variables coloured by contribution to PCs


fviz_pca_biplot(PCA.stress,
        axes = c(1,2),
        mean.point=F,             #
        axes.linetype = "solid",

        # Fill individuals by groups
        geom.ind=c("point", "text"),
        pointshape = 21,
        pointsize = 5,
        col.ind= "black",          #
        fill.ind = group.colors,
        alpha.ind = 1,             #

        # Color variable by intervals
        geom.var = "arrow",
        col.var = interval.colors,
        arrowsize = 0.5,

        repel = TRUE) +            # Avoid label overplotting


 #fill_palette(group.palette) +
 gradient_color(interval.palette)+
 labs(x = "PC1(51.66%)", y = "PC2(35.56%)")+


 theme(
   panel.grid.major = element_blank(),
   panel.grid.minor = element_blank(),
 )


#CONVERGENCE (example from simulations of pressure with the apical tooth only)

# 1) Read all the files included in the convergence

intervals.data.5 = read.csv("matrix-of-5-intervals_pressure_AT_logNN.csv",row.names=1, header = TRUE, sep = ",")
intervals.data.10 = read.csv("matrix-of-10-intervals_pressure_AT_logNN.csv",row.names=1, header = TRUE, sep = ",")
intervals.data.15 = read.csv("matrix-of-15-intervals_pressure_AT_logNN.csv", row.names=1,header = TRUE, sep = ",")
intervals.data.25 = read.csv("matrix-of-25-intervals_pressure_AT_logNN.csv",row.names=1, header = TRUE, sep = ",")
intervals.data.50 = read.csv("matrix-of-50-intervals_pressure_AT_logNN.csv", row.names=1,header = TRUE, sep = ",")

# 2) Compute PCA for each case
```

```
PCA.5 = prcomp(intervals.data.5[,1:5], scale=T)
PCA.10 = prcomp(intervals.data.10[,1:10], scale=T)
PCA.15 = prcomp(intervals.data.15[,1:15], scale=T)
PCA.25 = prcomp(intervals.data.25[,1:25], scale=T)
PCA.50 = prcomp(intervals.data.50[,1:50], scale=T)


# 3) Calculate te R-squared values for convergence procedure

# PC1 convergence:

Rvalues.pc1 = c(summary(lm(PCA.5$x[,1]~PCA.10$x[,1]))$r.squared,
  summary(lm(PCA.10$x[,1]~PCA.15$x[,1]))$r.squared,
  summary(lm(PCA.15$x[,1]~PCA.25$x[,1]))$r.squared,
  summary(lm(PCA.25$x[,1]~PCA.50$x[,1]))$r.squared)

# PC2 convergence:

Rvalues.pc2 = c(summary(lm(PCA.5$x[,2]~PCA.10$x[,2]))$r.squared,
  summary(lm(PCA.10$x[,2]~PCA.15$x[,2]))$r.squared,
  summary(lm(PCA.15$x[,2]~PCA.25$x[,2]))$r.squared,
  summary(lm(PCA.25$x[,2]~PCA.50$x[,2]))$r.squared)

# Table with results: R-squared values

data.pca = data.frame(Rvalues.pc1,Rvalues.pc2)
names(data.pca) = c("PC1","PC2")
rownames(data.pca) = c("PCA 5 vs. PCA 10", "PCA 10 vs. PCA 15","PCA 15 vs. PCA 25","PCA 25 vs. PCA 50")

# 4) PLot the PCAs and the R-squared values

plot(PCA.5$x[,1], PCA.5$x[,2], pch=19, cex=1.5, xlab = "", ylab ="", asp=T, main = "B) 5 intervals", xlim=rev(range(PCA.5$x[,1])) , ylim = (range(PCA.5$x[,2])))
plot(PCA.10$x[,1], PCA.10$x[,2], pch=19, cex=1.5, xlab = "", ylab ="", asp=T, main = "B) 10 intervals", xlim=rev(range(PCA.10$x[,1])) , ylim = rev(range(PCA.10$x[,2])))
plot(PCA.15$x[,1], PCA.15$x[,2], pch=19, cex=1.5, xlab = "", ylab ="", asp=T, main = "B) 15 intervals", xlim=rev(range(PCA.15$x[,1])) , ylim = rev(range(PCA.15$x[,2])))
plot(PCA.25$x[,1], PCA.25$x[,2], pch=19, cex=1.5, xlab = "", ylab ="", asp=T, main = "C) 25 intervals", xlim=rev(range(PCA.25$x[,1])) , ylim = rev(range(PCA.25$x[,2])))
plot(PCA.50$x[,1], PCA.50$x[,2], pch=19, cex=1.5, xlab = "", ylab ="", asp=T, main = "D) 50 intervals", xlim=rev(range(PCA.50$x[,1])) , ylim = rev(range(PCA.50$x[,2])))

plot(0,0,main = "F) Convergence", ylim = c(0, 1.2), xlim = c(1, 5))
points(Rvalues.pc1, col = c("tomato"), pch=19, cex=1.5)
points(Rvalues.pc2, col = c("salmon1"), pch=19, cex=1.5)
lines(Rvalues.pc1,lwd=1, col = c("tomato"))
lines(Rvalues.pc2,lwd=1, col = c("salmon1"))
abline(h=1, lty=3)
legend(4.3,0.5, legend= names(data.pca), pch=19,  col=c("salmon1","tomato"),cex=1)
```
